# Natural language processing in the classification of radiology reports in benign gallbladder diseases

**DOI:** 10.1590/0100-3984.2023.0096-en

**Published:** 2024-06-26

**Authors:** Lislie Gabriela Santin, Henrique Min Ho Lee, Viviane Mariano da Silva, Ellison Fernando Cardoso, Murilo Gleyson Gazzola

**Affiliations:** 1 Hospital Israelita Albert Einstein, São Paulo, SP, Brazil; 2 Universidade Presbiteriana Mackenzie, São Paulo, SP, Brazil

**Keywords:** Natural language processing, Neural networks, computer, Deep learning, Support vector machine, Artificial intelligence, Processamento de linguagem natural, Redes neurais de computação, Aprendizado profundo, Máquina de vetores de suporte, Inteligência artificial

## Abstract

**Objective:**

To develop a natural language processing application capable of automatically identifying benign gallbladder diseases that require surgery, from radiology reports.

**Materials and Methods:**

We developed a text classifier to classify reports as describing benign diseases of the gallbladder that do or do not require surgery. We randomly selected 1,200 reports describing the gallbladder from our database, including different modalities. Four radiologists classified the reports as describing benign disease that should or should not be treated surgically. Two deep learning architectures were trained for classification: a convolutional neural network (CNN) and a bidirectional long short-term memory (BiLSTM) network. In order to represent words in vector form, the models included a Word2Vec representation, with dimensions of 300 or 1,000. The models were trained and evaluated by dividing the dataset into training, validation, and subsets (80/10/10).

**Results:**

The CNN and BiLSTM performed well in both dimensional spaces. For the 300- and 1,000-dimensional spaces, respectively, the F1-scores were 0.95945 and 0.95302 for the CNN model, compared with 0.96732 and 0.96732 for the BiLSTM model.

**Conclusion:**

Our models achieved high performance, regardless of the architecture and dimensional space employed.

## Abstract (pt)

**Objetivo:**

Desenvolver uma aplicação de processamento de linguagem natural capaz de identificar automaticamente doenças cirúrgicas benignas da vesícula biliar a partir de laudos radiológicos.

**Materiais e Métodos:**

Desenvolvemos um classificador de texto para classificar laudos como contendo ou não doenças cirúrgicas benignas da vesícula biliar. Selecionamos aleatoriamente 1.200 laudos com descrição da vesícula biliar de nosso banco de dados, incluindo diferentes modalidades. Quatro radiologistas classificaram os laudos como doença benigna cirúrgica ou não. Duas arquiteturas de aprendizagem profunda foram treinadas para a classificação: a rede neural convolucional (*convolutional neural network* - CNN) e a memória longa de curto prazo bidirecional (*bidirectional long short-term memory* - BiLSTM). Para representar palavras de forma vetorial, os modelos incluíram uma representação Word2Vec, com dimensões variando de 300 a 1000. Os modelos foram treinados e avaliados por meio da divisão do conjunto de dados entre treinamento, validação e teste (80/10/10).

**Resultados:**

CNN e BiLSTM tiveram bom desempenho em ambos os espaços dimensionais. Relatamos para 300 e 1000 dimensões, respectivamente, as pontuações F1 de 0,95945 e 0,95302 para o modelo CNN e de 0,96732 e 0,96732 para a BiLSTM.

**Conclusão:**

Nossos modelos alcançaram alto desempenho, independentemente de diferentes arquiteturas e espaços dimensionais.

## INTRODUCTION

Benign gallbladder diseases are highly prevalent and occur because of a variety of etiological factors, including cholelithiasis, microlithiasis, adenomyomatosis, polyposis, and cholesterolosis. The most notorious of those is cholelithiasis, which can be defined as the presence of calculi within the gallbladder (gallstones), reportedly affecting approximately 6.3 million men and 14.2 million women between the ages of 20 and 74 in the United States^(1)^. Most individuals with gallstones are asymptomatic and are diagnosed through routine imaging tests or during the investigation of other abdominal diseases. Many such individuals remain asymptomatic and do not require treatment. However, when indicated, a cholecystectomy is performed, and though definitive, it is not risk-free^(2)^. Therefore, the surgical indication ought to show specific criteria. In this context, it is necessary to evaluate radiology reports in order to extract information about gallbladder diseases. Conducted manually and individually, this assessment is labor-intensive, particularly if done on a large scale. In order to solve that problem, natural language processing (NLP) has been used. An interdisciplinary applied research field encompassing computer science and artificial intelligence that analyzes natural language data, NLP serves as an intersection between computer science and linguistics, with the aim of developing decision support systems.

Clinical decision support systems are defined as any software designed to directly assist in clinical decision making, in which the characteristics of each patient are considered together with data in a knowledge base. The aim is to generate specific assessments or recommendations that are presented to professionals for their consideration. In advanced models, for example, those can include the determination of drug interactions, the identification of diseases, individualized dosage support in cases of renal failure, and recommendations for laboratory tests during pharmacological treatment^(3)^.

The use of NLP methods provides a means for people to work using their natural language and still be able to develop algorithms that manipulate, augment, and transform natural language into a computable format. Therefore, NLP has proven effective in extracting information from radiology reports, including the detection of critical findings and quality assessment, as well as the generation of annotations and datasets^(4)^, making it the ideal method for the development of an algorithm like the one developed in the present work.

## MATERIALS AND METHODS

To build a database for this project, we first compiled 1,100 radiology reports that described changes in the gallbladder, all for examinations performed at our hospital in January or February of 2018. We also included 100 reports of randomly chosen patients who underwent clinical follow-up for cholecystectomy in the same period, bringing the total number of reports in the database to 1,200. The scope of the reports encompasses three different types of examinations, the distribution of which is shown in Table 1. We divided the report dataset into two groups of 600 reports, aiming at the subsequent stage of annotations made by radiologists.

**Table 1:** Types of reports used and their distribution in the annotation groups.

Type of report	Group 1	Group 2
Ultrasound	318	300
Computed tomography	201	204
Magnetic resonance imaging	81	96
Total	600	600

### Preprocessing

The correct identification of terms when using an NLP model with named entity recognition requires sentences that are standardized, single, and complete sentences. Given that need, we observed that some of the reports contained sentences that were not configured correctly-with line breaks in the middle of the sentence, multiple sentences in the same line, and special cases of punctuation for dividing sentences.

The full report content made available was divided into lines, used in order to train the sentence model. The lines were labeled as belonging to one of four classes: correct, single sentences; single sentences with inappropriate breaks; complete, multiple sentences; multiple sentences with breaks.

The data preprocessing phase, designated data cleaning, comprises a total of four main steps. The first data processing step consists in converting all text to lowercase letters, followed by the removal of punctuation and stop words. Subsequently, the corpus undergoes two conversions-bag of words and term frequency-inverse document frequency, techniques employed for quantitative and numerical representation, respectively. In addition, sentences containing sensitive data, pagination information, or instructions for accessing the report were removed, as well as the signature of the radiologist. Said sentences, which are present in all reports, do not influence the clinical findings in any way. Therefore, those portions of the text are removed only to help reduce the size of the dataset.

### Report annotation

Radiology reports consist of several findings relating to the physiological and structural conditions of the organs, though not all information directly contributes to the final diagnosis. The need for physician intervention can be identified from the presence of certain expressions or sentences, related to the selected labels (Table 2). All the labels were coded according to the RadLex, a specific ontology used in radiology^(5)^.

**Table 2:** Key terms noted in the reports.

RadLex code	Term
RID187	Gallbladder
RID3394	Cholecystitis
RID34607	Microlithiasis
RID3869	Adenomyomatosis
RID3881	Polyp
RID4989	Gallstone
RID5198	Porcelain gallbladder
RID5215	Cholesterolosis

**Figure 1:**
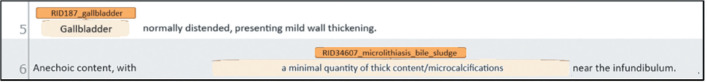
Annotation environment on the INCEpTION platform, with some entities already classified by the radiologist.

**Figure 2:**
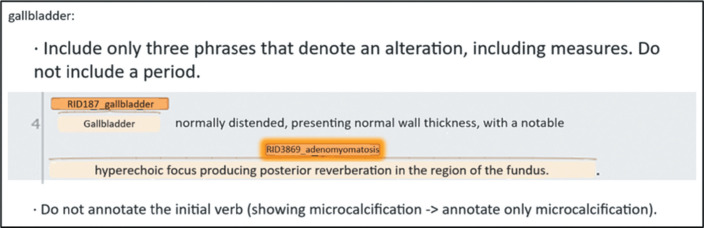
Examples of rules for resolving questions, described in the manual for the standardization of annotations.

All documents were anonymized and stored in an environment, within the hospital system, that was secure and dedicated to annotation. Three radiologists and one radiology resident analyzed the documents and identified the established terms, indicating their position and sentence of the occurrence. The INCEpTION platform (Figure 1) was used by each specialist for individual annotations, and each annotator had a specific login and password. The INCEpTION platform also allowed comparisons between the different findings in each document, to define the final terms of the report. Given the limited experience that radiologists have with such annotation work, a manual was prepared, with clear guidelines on logging on to the INCEpTION platform, which words to include in the annotation, and how to correctly classify the terms (Figure 2), aimed at answering any questions and standardizing the data for better model training.

In addition to the annotation of clinical terms, the reports were classified according to the presence of indications for cholecystectomy. To that end, a board of specialists from the hospital prepared a compliance and noncompliance checklist for cholecystectomy indication (Table 3), based on international guidelines and those of the hospital itself^(6,7)^. The checklist includes cholecystectomy indications in clinical and surgical practice, which are used for labeling cases as appropriate or inappropriate for surgical indication.

**Table 3:** Classification table of compliance and noncompliance in the indication of cholecystectomy, prepared by experts.

Aspects of compliance	Aspects of possible non compliance
Gallstone	Adenomyomatosis without associated symptoms or criteria
Microlithiasis	Cholesterolosis without associated symptoms or criteria
Bile sludge/thick bile	Report mentioning no changes in the gallbladder
Cholelitiasis/cholecistitis	Lack of examinations
Polyp > 5 mm	-
Porcelain gallbladder	-

### Curation

After the reports had been annotated, a curation phase was carried out to establish a gold standard corpus. In that phase, the annotations were compared between the pairs, resolving the points of divergence identified through consensus among the participants. Agreement between annotators was assessed using Cohen’s kappa coefficient of agreement, which allows the complexity of the task and the precision with which the annotation was made to be assessed. A kappa value ≥ 0.81 is considered indicative of perfect agreement^(8)^. Kappa values of 0.97 and 0.88 were obtained for group 1 and group 2, respectively. The annotations resulting from the curation phase were taken as the gold standard for the training and testing of the models.

### Training

For training, we used multiple techniques. One such technique is word embedding, which transforms words into vectors of real numbers, representing them in an n-dimensional space (typically 300-dimensional). Another one is learning from large unannotated corpora, which allows syntactic, semantic, and morphological knowledge to be captured, thus enabling the efficient “translation” of texts, and which has become commonplace in NLP systems.

There are several different word embedding tools available, including Word2Vec, which was one of the first to gain popularity^(9)^. The Word2Vec technique uses a two-layer neural network that processes text through the vectorization of words. Its input is a corpus of text, and its output is a set of vectors, one for each word. Those vectors group similar words together, allowing the mathematical similarity of the vectors to be determined through the use of cosine similarity^(9)^. With sufficient, correct data and text, Word2Vec can make highly accurate assumptions about the meaning of a word based on its occurrence in the corpus, which is why this method was chosen to train our model.

Deep learning algorithms were adopted, with a convolutional neural network (CNN) architecture, adapted for text, that was the first one used. The structure of this CNN included two convolutional layers, containing 64 and 128 filters with convolutional dimensions of 7 and 5, respectively. Both convolutional layers used the tanh activation function, followed by max pooling and the inclusion of two other dense layers with ReLU activation, mediated by a dropout layer. The optimizer used was Adam, with a learning rate of 0.0001.

Recurrent neural network architectures, especially long short-term memory (LSTM) networks, are also widely used in the context of NLP. The bidirectional variation of this type of architecture (BiLSTM) was used for training. Two BiLSTM layers, containing 256 and 128 neurons, respectively, were mediated by an attention layer with sigmoid activation. A dense layer, with ReLU activation, was also included after the second BiLSTM layer. In both types of architecture, a word embedding layer was included for text vectorization. In the English language, there are several natural language processing repositories aimed at the health segment; biomedical named entity recognition is one example^(10)^. In Portuguese, however, there were difficulties because there was no specific database. Therefore, in our experiments, we employed the word embeddings made available in Brazilian Portuguese by the Brazilian Interinstitutional Center for Computational Linguistics^(11)^, with 300-dimensional and 1,000-dimensional vectors.

The training was conducted on a computer with an Intel Core i5-10210U CPU @ 1.60 GHz 2.10 GH and 16 GB RAM. The programming language used was Python, version 3.8.

## RESULTS

Validation of the performance of the CNN and BiLSTM models was performed by separating the dataset into training, validation, and test subsets, in proportions of 80%, 10%, and 10%, respectively. The CNN models with lemmatization presented F-scores of 0.95945 and 0.95302 for the Word2Vec embeddings with 300-dimensional and 1,000-dimensional vectors, respectively. The BiLSTM recurrent CNN architectures with lemmatization reached F-scores of 0.96732 and 0.96732, respectively, for those same Word2Vec embeddings (Table 4).

**Table 4:** F-scores for the CNN and BiLSTM models.

Model	F-score (80/10/10)
CNN + lemmatization + Word2Vec 300d	0,95945
CNN + lemmatization + Word2Vec 1000d	0,95302
BiLSTM + lemmatization + Word2Vec 300d	0,96732
BiLSTM + lemmatization + Word2Vec 1000d	0,96732

80/10/10, proportional distribution of the training, validation, and test subsets, respectively; 300d, 300-dimensional vector space; 1,000d, 1,000-dimensional vector space.

## DISCUSSION

Among the distinct types of deep learning architectures, CNNs and recurrent neural networks are the ones that have been trained for the task of classification. In both cases, a Word2Vec embedding layer, pre-trained with a Portuguese-language corpus, was allocated to the input to represent the text in vector form. The word embedding was alternated between 300 and 1,000 dimensions in order to determine its influence on the results.

The metrics obtained through validation of the different models show that they are similar in performance. Increasing the dimensions in the Word2Vec representation does not seem to have a significant impact, given that the results were similar for both dimensionalities. One possible explanation for that behavior is the closed scope of the evaluation of a specific anatomical structure and of surgical treatment of benign diseases, which severely limits the number of words contained in a sentence and their variations. Radiology reports have specific technical descriptors to facilitate communication among health care professionals, and that shortens the length of these medical texts.

Although we obtained positive results, it is crucial to identify and analyze the limitations that could affect the interpretation and application of our results. The first limitation is the origin of the reports, which were obtained from a single hospital. This limits representativeness, because language and clinical approaches can differ among health care facilities. In addition, the reports were not originally intended for training the algorithm but rather for diagnosing diseases in the clinical routine. That can affect the data quality because the reports might not present the diversity required for comprehensive training, providing limited insight into the applicability of the algorithm, especially in clinical scenarios that are less common or more complex. The decision to focus only on benign gallbladder diseases was made due to restrictions regarding the complexity associated with evaluating medical records and confirming the diseases diagnosed. However, that choice could limit the applicability of the algorithm in cases of rarer and more complex diseases, thus reducing the generalizability of the results. By acknowledging and discussing these limitations in a transparent manner, we contribute to the integrity of the study and promote the ongoing advancement of the application of algorithms in clinical settings.

## CONCLUSION

Gallbladder diseases are quite prevalent in the population, and their treatment commonly involves a surgical procedure, which, despite rarely presenting complications, is not without risk. To ensure that the indication for surgery followed precise criteria, we developed a prototype of a clinical decision support system that extracts information from radiology reports, classifying gallbladder diseases as requiring or not requiring surgical treatment. To facilitate this process, we employed NLP. That allows the automation of a variety of tasks in radiology and is a valuable area of research in the analysis, aggregation, and simplification of unstructured (textual) data, having already demonstrated significant potential in the analysis of radiology reports.

The wide availability of numerous open source libraries and tools facilitates their application for the benefit of radiology. Radiologists who understand their limitations and potential will be better positioned to evaluate NLP models, understand how they can improve clinical workflow, and facilitate research efforts involving large amounts of human language. There is also significant potential for the field of radiology to benefit from the ability of NLP to convert radiology reports into machine-readable data.

Our models achieved high performance in using NLP to automatically identify and extract data regarding the presence or absence of benign gallbladder diseases requiring surgery from radiology reports, regardless of the architecture and dimensional space employed. These approaches can be extended to other clinical scenarios by using a similar method to extract and structure information from large datasets.

## References

[1] Afdhal NH, Zakko SF. Gallstones: epidemiology, risk factors and prevention.

[2] Zakko SF. Overview of nonsurgical management of gallbladder stones.

[3] Wasylewicz ATM, Scheepers-Hoeks AMJW., Kubben P, Dumontier M, Dekker A (2019). Fundamentals of clinical data science.

[4] Bressem KK, Adams LC, Gaudin RA (2021). Highly accurate classification of chest radiographic reports
using a deep learning natural language model pre-trained on 3.8 million text
reports. Bioinformatics.

[5] RSNA RadLex radiology lexicon.

[6] Sociedade Beneficente Israelita Brasileira Colecistectomia laparoscópica.

[7] Soper NJ. Laparoscopic cholecystectomy.

[8] Landis JR, Koch GG. (1977). The measurement of observer agreement for categorical
data. Biometrics.

[9] Hartmann NS. (2020). Adaptação lexical automática em textos informativos
para o ensino fundamental.

[10] Wang K, Zhang Y, Ren S Cross-type biomedical named entity recognition with deep multi-task
learning.

[11] Repositório de Word Embeddings do NILC.

